# A Case of Compound Comminuted Fracture of the Frontal Bone, with Depression, Recovery

**Published:** 1886-11

**Authors:** Eugene Clark

**Affiliations:** Lockhart, Texas


### A CASE OF COMPOUND-COMMINUTED FRACTURE OF THE FRONTAL BONE, WITH DEPRESSION: RECOVERY.

*By Eugene Clark, M. D., Lockhart, Texas.*

For Daniel's Texas Medical Journal.

ON the evening of June 4th, last, I was called to see a negro boy aged about 18 years, who had been hit in the forehead a few hours before my arrival, with a rock about the size of two fists. When hit, he fell as though dead; was picked up and brought to the house, a distance of three hundred yards, after which he slowly revived. When I arrived, I found him lying in a semi-conscious condition; pulse slow, full and regular; respiration slow, labored and irregular; pupils dilated. There was a transverse gash about two inches long, an inch above the eye brows This communicated with a depressed and comminuted fracture of the frontal bone. It being night, I dressed the wound, made the patient as comfortable as possible, and postponed further proceedings until daylight.

When seen the next day, the patient complained of pain and heaviness in the head; articulation indistinct, pulse full and regular, breathing slow and irregular, pupils dilated. He was put under the influence of a mixture of chloroform and ether, the cut enlarged and an incision made at right angle and from the middle of the upper edge of the first cut. The skin was dissected back revealing a depressed triangular fracture, the sides of which were about two and a half inches long. The base of the triangular fracture was downward, and was badly comminuted. The comminuted bone was removed, and an elevator introduced into the opening made by its removal, and the depressed portion of bone elevated into position. In removing this comminuted bone, there was one spicula of the internal table which had perforated the dura mater and entered the longitudinal sinus, upon the removal of which there came forth a continuous stream of black blood about an eighth of an inch in diameter. The longitudinal incision was closed with sutures, and a piece of absorbent cotton introduced to check the hemorrhage from the opened sinus. The transverse cut was left open for drainage, and the removal of the cotton. The wound was dressed with a dry dressing of absorbent cotton.

Upon coming from under the influence of the anæsthetic, he was able to get up from the table and walk to his bed. He said that his head felt free from heaviness or pain, and his speech was clear and distinct.

When seen on the 6th, the temperature was 98½°, pulse 87, respiration 22, pupils normal, intellect clear. The cotton which had been introduced to check the hemorrhage from the opened sinus, was removed, and there was no further trouble from hemorrhage. The wound was washed out with a disinfectant solution and dressed as before. with a dry dressing. The patient did well—at no time did the temperature go over 101°. On the 12th the patient sat up and shelled corn. In four weeks time the wound had healed, leaving the patient, with the exception of a slight scar, none the worse for the mishap.

---

CINCINNATI, OHIO, September 24, 1886.

F. E. DANIEL, M. D., Austin, Texas.

*Dear Doctor:*— * * * You can rest assured I will give you whatever business I can for your publication, as I regard it as one of the most enterprising of the many Medical Journals of the South. You have certainly made great headway with it.

Yours very truly, H. C. HALL.

